# A Cross-sectional Analysis of Glove Perforation in Primary and Revision Total Hip Arthroplasty

**DOI:** 10.5704/MOJ.1611.010

**Published:** 2016-11

**Authors:** D Kumar, K Shantanu, M Kumar, A Kumar, V Sharma

**Affiliations:** Department of Orthopaedics, King George’s Medical University, Lucknow, India

**Keywords:** Surgical gloves, perforation, primary total hip arthroplasty, revision total arthroplasty, latex

## Abstract

The number of total hip arthroplasties is ever increasing. Literature about glove perforation rates in arthroplasties in India is very scarce. The purpose of our study was to determine the incidence of glove perforation and increasing the awareness of possible glove perforations to decrease the risk of infection. We performed a prospective study in which we tested gloves worn by all scrubbed personnel. A total of 1408 gloves were collected from 42 primary total hip and 13 revision total hip arthroplasties. Incidence of glove perforation was found to be more in revision total hip arthroplasty. We found a greater outer glove perforation rate of about 38.33% as compared to 25 % inner glove perforation rate. Outer glove perforation was recognized 100% of time intraoperatively but inner glove perforation was noted only 17% of time. First assistant recorded highest rate of glove perforation.

## Introduction

Total hip arthroplasty is a very rewarding surgery. As the number of total hip arthroplasties is increasing day by day^1^, revision total hip surgeries are also increasing. In the Western literature infection rates are found to be 0.88% to 0.92% for primary arthroplasty and 2.9% to 30.2% for revision arthroplasty^1-7^. Periprosthetic infections are among the most devastating complications for the patients as well as for the surgeons. The etiology of periprosthetic infections is multifactorial. Diabetes and poor socioeconomic status are the major patient related factors, while major surgical factors include operative time and allogenic blood transfusion whereas postoperative complications such as urinary tract infection, myocardial infarction and longer duration of hospitalization have also been attributed to increased risk of periprosthetic infections^8^. Laminar air flow and prophylactic antibiotics are commonly used by the surgeons to decrease the risk of perioperative infections^8-10^. Despite all the efforts made by surgeons strict aseptic technique is essential to minimize the risk of surgical field contamination.

The reported incidence of glove perforation in orthopaedic procedures is found to be between 3.6% and 26%^11-18^. Previous studies have shown an association between glove perforation and duration of procedure, hand dominance, and specific portions of procedure^11,12,14,19^. The present authors have not found any studies in India that specifically evaluate incidence of glove perforation in primary and revision total hip arthroplasty.

This cross-sectional, comparative study was designed to evaluate the incidence of glove perforation in primary and revision total hip arthroplasty. The main objective was to compare the incidence of glove perforation in primary and revision total hip arthroplasty. The secondary objective was to study the factors responsible for glove perforation in total hip arthroplasty and to increase the awareness of surgeons to glove perforation in total hip arthroplasty. We hypothesized that the rate of glove perforation is greater in revision total hip as compared to primary total hip arthroplasty.

## Materials and Methods

This study was conducted during the period from June 2014 to May 2015 at our institute. Natural rubber latex, textured and non-powdered gloves (B.Braun Medical Ltd.) which is routinely provided by the University, were used in the study. A single arthroplasty team of surgeons performed all the surgeries to maintain the same techniques and standards during surgery. We followed triple-gloving protocol for all of surgeries in the study. The outermost (preparation) layer used for draping purposes and then discarded. The second layer was worn throughout the procedure and changed as and when required. The first innermost layer was worn right through unless a perforation was noted.

Usually a team of five or six personnel were scrubbed in a procedure. We collected all the data regarding scrubbed personnel before each procedure such as role of the person during the case and hand dominance, after having obtained an informed consent from all. At the end of each procedure we collected all the gloves, labeled individually and placed in marked plastic bags with details of the person who had worn those gloves. At time of gloves removal, data such as glove material, layer, side, duration of wear, time of removal and reason of glove removal were recorded.

**Fig. 1:**
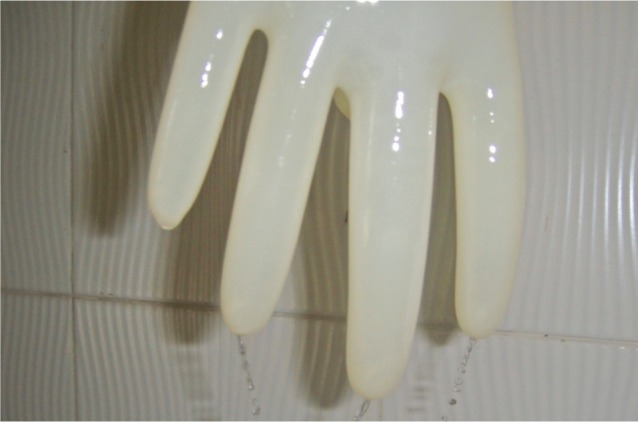
Identification of perforations by pressurization of the gloves filled with water.

At completion of each procedure all the collected gloves were reexamined for perforations using standardized water infusion method described by the American Society for Testing and Material Guidelines^20^. We tested the gloves by filling them with 1000ml of water and suspended from the occluded cuff, 5ft from the ground (Fig. 1) .The gloves and the digits were pressurized and all the perforations were identified by a jet of water^20^. Perforation were noted as per their location, size, number and cause.

Statistical analysis of data was conducted to evaluate significant association with the factors analyzed and perforations using the Fisher exact test. P value less than .05 were considered statistically significant.

## Result

All the gloves were collected from 42 primary and 13 revision total hip arthroplasties performed at our institute during the period of study. A total one thousand four hundred and eight gloves were retrieved from all the scrubbed up personnel. The total incidence of glove perforation was found to be 6.25% (88/1408) if all gloves were included. We examined the gloves of the chief surgeon, first assistant, second assistant and scrub nurse. The number of perforations related to glove layer and seniority of scrubbed personnel are listed in Table I. Outer glove perforations were noticed in 38.33%(23/60) whereas inner glove perforations were noticed only 25% (5/20).Total glove perforations of the chief surgeon was noted to be 6.06%(18/297). Out of all scrubbed personnel maximum glove perforations encountered were with the first assistant 10.9% (45/413) and that was inclusive of all three layers. Probably the reason behind this was that the assistant was involved in performing the parts of the procedure most at risk to glove perforation. The first assistant had a 14% (42/300) combined outer and inner glove perforation rate in all cases. Outer gloves perforated in 16.38% (29/177) and inner gloves in 10.57% (13/123).

**Table I:** Rates of glove perforation according to scrubbed personnels

Particular	Perforation	%	Non Perforated	%	Total used
	**Chief Surgeon**				
Prep gloves	1	2.38	41	97.62	42
Outer gloves	15	10.48	128	89.52	143
Inner gloves	2	1.78	110	98.22	112
	**First Assistant**				
Prep gloves	3	2.65	110	97.35	113
Outer gloves	29	16.38	148	83.62	177
Inner gloves	13	10.57	110	89.43	123
	**Second Assistant**				
Prep gloves	2	1.79	110	98.21	112
Outer gloves	10	7.81	118	92.19	128
Inner gloves	5	4.35	110	95.65	115
	**Scrub Nurse**				
Prep gloves	2	1.79	110	98.21	112
Outer gloves	6	5.41	105	94.59	111
Inner gloves	0	0	110	100	110

Comparison of incidence of glove perforation in primary and revision THA is presented in Table II. Total glove perforations occurred in 5.24% (57/1087) worn during primary THA compared with 9.65% (31/321) in revision THA. The inner layer was perforated in 4.34% (20/460) of all THA cases. Outer gloves had an incidence of 10.73% (60/559) of all THA cases. The first assistant had outer glove perforation in 8.67% (28/323) of primary THA compared with 18.89% (17/90) in revision cases (P=.01).

**Table II:** Outer gloves perforations: Primary vs Revision THA

Particular	Perforation	%	Non perforated	%	Total used	p value
**Chief Surgeon**						
Primary THA	13	5.49	224	94.51	237	0.689
Revision THA	5	7.81	59	92.19	64	
**First Assistant**						
Primary THA	28	8.67	295	91.13	323	0.01
Revision THA	17	18.89	73	81.11	90	
**Second Assistant**						
Primary THA	10	3.65	264	96.35	274	0.148
Revision THA	7	8.24	78	91.76	85	
**Scrub Nurse**						
Primary THA	6	2.37	247	97.63	253	0.972
Revision THA	2	2.44	80	97.56	82	

In primary and revision THA cases, total 75.75 % (25/33) of perforations occurred from exposure to preparation of bone (Table III). Total of 88 perforations were found in all the collected gloves out of which only 37.5 % (33/88) had been noticed during the procedure and gloves changed accordingly. Most of the perforations noticed in the preparation layer and outer layer of gloves 31.81% (28/88). Inner layer perforations were occasionally noticed (Table IV).

**Table III:** Glove perforations noticed according to stage of operation

	Number	%
Setting up	2	6.06
Preparation of bone	25	75.75
Closure	6	18.19
Total	33	

**Table IV:** Total number of gloves perforations and awareness of perforations

Combined	Perforations	Noticed	%
Prep gloves	8	5	62.5
Outer gloves	60	23	38.33
Inner gloves	20	5	25.00
Total	88	33	37.50

Analysing the time duration of wearing of the gloves there was no significant difference between perforated and nonperforated gloves in the group of scrubbed personnel. The mean time recorded was (53+-17min). Sixty three percent perforations were found on the index finger, followed by 20% on the thumb, while most of the (76%) perforations occurred on the non-dominant hand.

## Discussions

During any surgical procedure gloves act as a vital component in maintaining the barrier between surgical team and the patient. Gloves reduce the chances of risk of disease transmission and subsequent infection^21-24^. For this reason glove perforation is a serious complication that needs to be addressed vigorously because it exposes both the surgical team as well as the patient to the risk of infection. This study is the first of its type in the Indian setup to demonstrate the incidence of glove perforation in primary as well as revision THA. Furthermore, this study is important to enhance the awareness of glove perforation as sufficient number of perforations were not noticed by the surgical team in most of the cases.

We used a double gloved protocol in all the cases while a third (outer) layer of gloves were used for draping and preparation (preparation layer). Overall 6.25% gloves were found to be perforated among all the scrubbed personnel. Perforations were recorded in outer gloves in 10.73% and 4.34% perforations were found in the inner gloves. The reported incidence of glove perforations in the Western literature ranges from 6.8% to 14.6% in THA and TKA ^17,25^.

We found a significant increase in the incidence of glove perforations of the first assistant during revision THA as compared to primary THA and this was mainly because of the more complex procedures with increased duration of surgery and greater exposure to sharp bone and metal.

This study was conducted with many limitations. The foremost limitation was of unnoticed perforations; however, the scrubbed persons had changed the gloves time to time but it was still not possible to detect every unnoticed perforation. The second limitation was of the water infusion method that was used to detect perforations. This method used to overdistend the gloves, causing water to come out through the perforation in jet form. In this method the main concern was that pressurization might have caused perforations in the gloves where structural integrity was probably compromised but actual perforation had not existed before testing.

There was no significant difference between the wearing time duration of perforated and non-perforated gloves which was probably because of routine changing of gloves at different stages of the procedure. However two studies reported previously that the incidence of glove perforations increases with duration of glove wear^26^. Most perforations occurred on the index finger of non-dominant hand, these findings are supported by previous studies as well.

## Conclusion

Increasing the awareness for glove perforations is very vital in preventing disease transmission or contamination between patient and operating room personnel. Most of the glove perforations go unnoticed, so a practical guideline for wearing the gloves, duration of glove wearing and assessment of glove perforation should be formulated. Orthopaedic procedures especially arthroplasties bear a higher risk of glove perforations, more so with revision surgeries. Therefore when a perforation is detected removal and a careful inspection of inner layer before regloving with a new outer layer is required.
